# Supplementary material for: Whole-genome sequencing analysis of anthropometric traits in 672,976 individuals reveals convergence between rare and common genetic associations
Source: Nat Commun. 2026 Feb 6;17:2432. doi: 10.1038/s41467-026-69208-3 (PMC12987921; doi:10.1038/s41467-026-69208-3)
Supplement: Supplementary file 1 — Supplementary Information [file 41467_2026_69208_MOESM1_ESM.pdf]

Supplementary Information

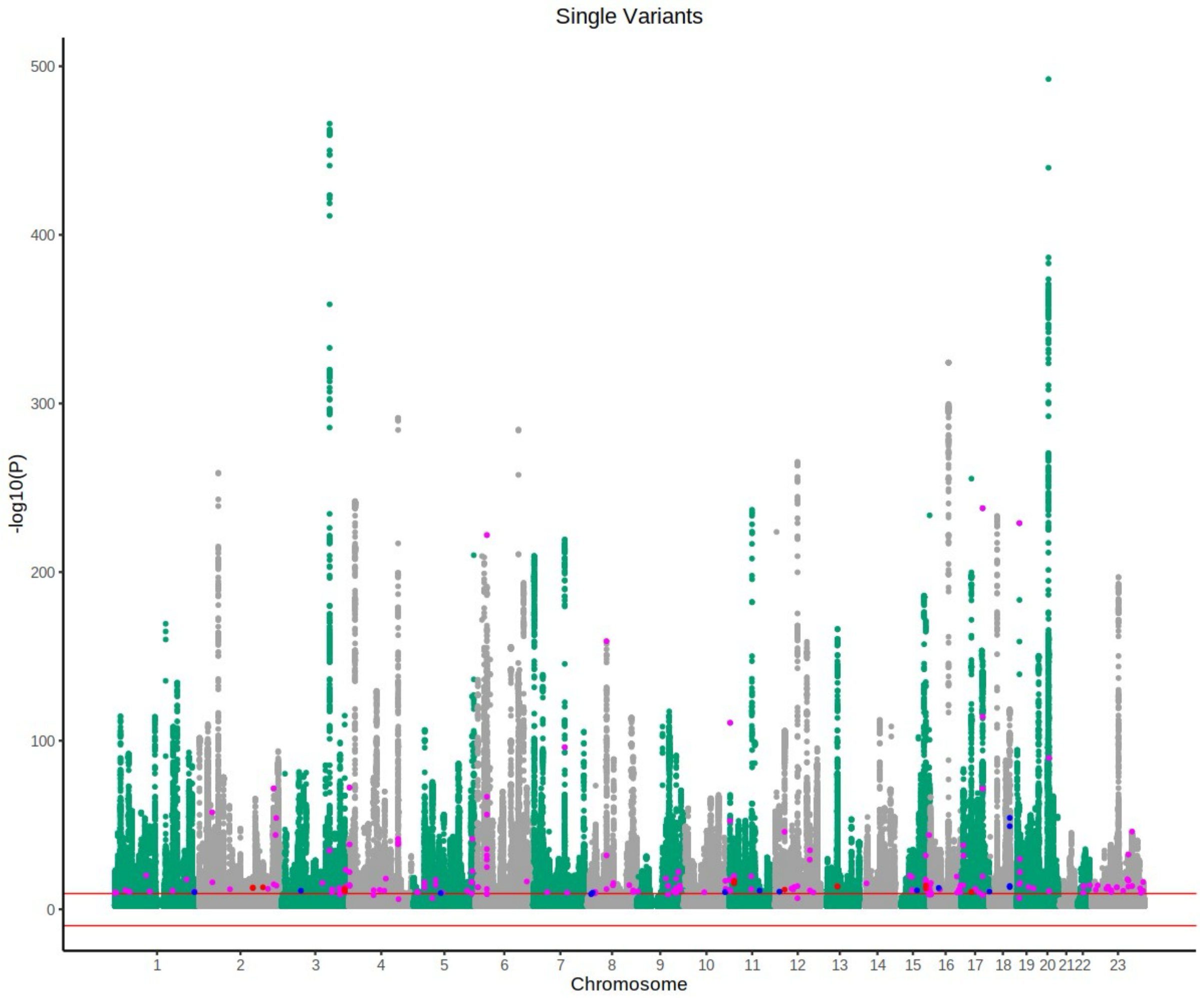

Supplementary Figure 1: Manhattan plot of single variant associations with anthropometric traits in UKB. Summary of all single variant associations for height, BMI and WHRadjBMI: the x-axis represents genomic position, segregated by chromosome and represented by alternating green and silver colours, on the x-axis, and -log10(P) on the y-axis. Jointly independent significant variants for each trait are uniquely coloured: magenta for height, red for WHRadjBMI and blue for BMI.

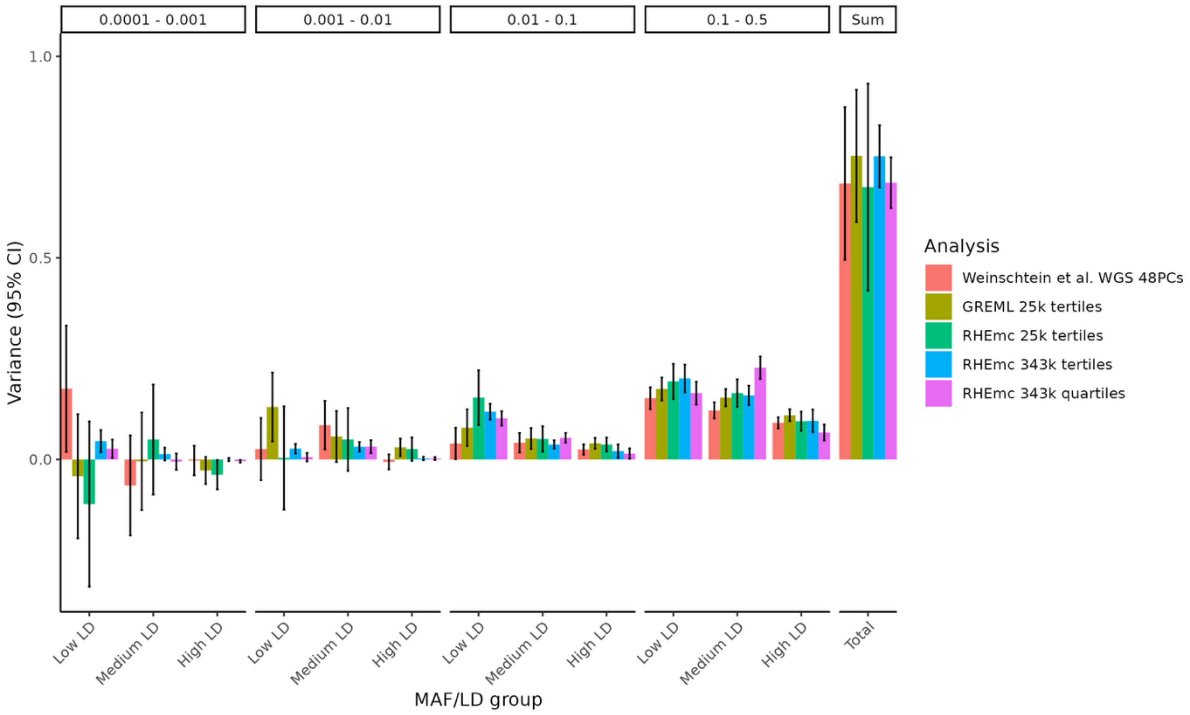

<u>Supplementary Figure 2: WGS height Heritability comparisons with Wainschtein *et al* (2022)</u>. Estimated heritability within each UKB MAF and LD-score strata, as determined by four different analyses across two methods (blue: RHEmc 343k LD tertiles, purple: RHEmc 343k LD quartiles, green: RHEmc 25k LD tertiles, dark yellow: GREML 25k LD tertiles) in comparison with *Wainschtein et al (2022)* who used TOPMed data.

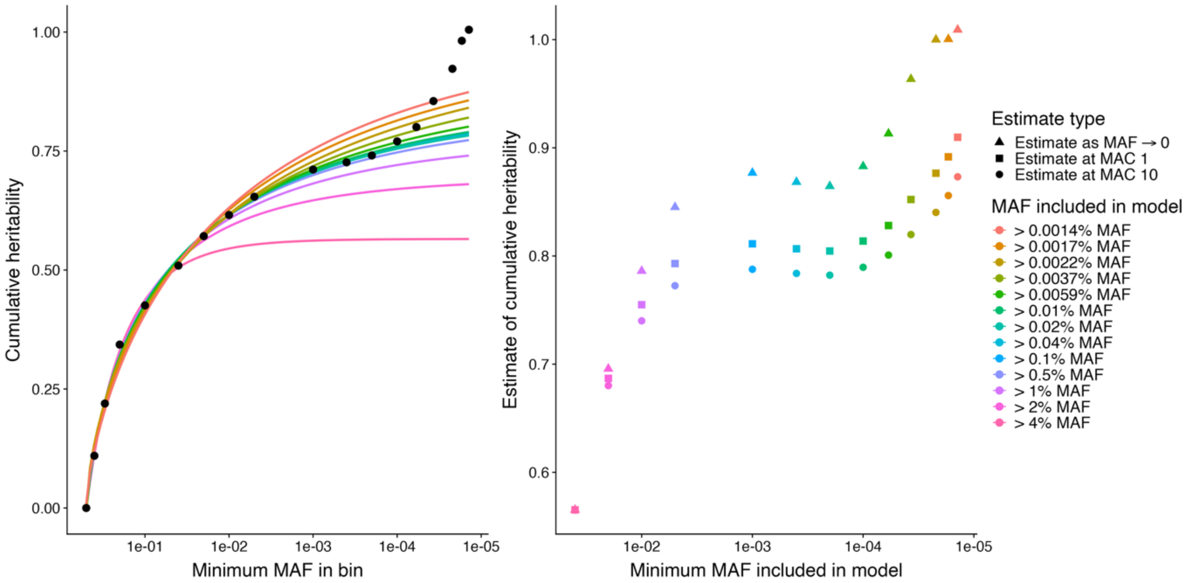

Supplementary Figure 3: Estimating population stratification for heritability analysis. Left: Fitted exponential decay models ($y = a - \exp(-b*(-\log_{10}(x) + c)^d)$) for the relationship between minor-allele-frequency bin and cumulative heritability. Each model prediction (coloured line) is built from the inclusion of heritability estimates (black points) from progressively lower MAFs (see legend). **Right**: estimates of cumulative heritability predicted by exponential decay models (coloured by model) at three MAC/MAFs (point shapes)
