# Supplementary material for: Whole-genome sequencing analysis of anthropometric traits in 672,976 individuals reveals convergence between rare and common genetic associations
Source: Nat Commun. 2026 Feb 6;17:2432. doi: 10.1038/s41467-026-69208-3 (PMC12987921; doi:10.1038/s41467-026-69208-3)
Supplement: Supplementary file 2 — Description of Additional Supplementary Files [file 41467_2026_69208_MOESM2_ESM.docx]

**Description of Additional Supplementary Files**

| Supplementary Data 1 | Single variant associations identified using CoJo for BMI, height and WHRadjBMI |
| --- | --- |
| Supplementary Data 2 | Single variant associations (ST1) significant after adjusting for known GIANT loci |
| Supplementary Data 3 | Single variant replication association results from All of Us |
| Supplementary Data 4 | Single variant imputation qualities and availability from the HRC+UK10K, TOPMed and Genomics England (GEL) imputation panels |
| Supplementary Data 5 | *IGF2BP2-*WHRadjBMI single variants phewas association results |
| Supplementary Data 6 | Conditionally independent coding rare-variant aggregate associations for BMI, height and WHRadjBMI |
| Supplementary Data 7 | Conditionally independent coding rare-variant aggregate associations for BMI, height and WHRadjBMI adjusted for known GIANT loci |
| Supplementary Data 8 | Replication in All of Us for conditionally independent coding rare-variant aggregate associations for BMI, height and WHRadjBMI |
| Supplementary Data 9 | Conditionally independent non-coding rare-variant aggregate associations for BMI, height and WHRadjBMI |
| Supplementary Data 10 | Conditionally independent non-coding rare-variant aggregate associations for BMI, height and WHRadjBMI adjusted for known GIANT loci |
| Supplementary Data 11 | Replication in All of Us of conditionally independent non-coding rare-variant aggregate associations for BMI, height and WHRadjBMI |
| Supplementary Data 12 | Statistically significant coding rare-variant aggregate associations for BMI, height and WHRadjBMI in the UKB-meta analysis |
| Supplementary Data 13 | Statistically significant non-coding rare-variant aggregate associations for BMI, height and WHRadjBMI in the UKB-meta analysis |
| Supplementary Data 14 | MAC>5 single variant associations contributing to the *FGF18*-Height 5'UTR aggregate association |
| Supplementary Data 15 | Proximity of single-variant genetic associations identified in UKB EUR-based analysis to 12,111 previously reported common SNP associations. Genetic associations shown remained genome-wide significant after adjustment for the 12,111 SNPs and reached P<0.05 in AoU-EUR analysis. |
| Supplementary Data 16 | Proximity of aggregate-based genetic associations identified in UKB EUR-based analysis to 12,111 previously reported common SNP associations. Genetic associations shown remained genome-wide significant after adjustment for the 12,111 SNPs and reached P<0.05 in AoU-EUR analysis. |
| Supplementary Data 17 | Proximity of single-variant genetic associations for BMI identified in UKB EUR-based analysis to 941 previously reported common SNP associations. Genetic associations shown remained genome-wide significant after adjustment for the 941 SNPs and reached P<0.05 in AoU-EUR analysis |
| Supplementary Data 18 | Proximity of aggregate-based genetic associations for BMI identified in UKB EUR-based analysis to 941 previously reported common SNP associations. Genetic associations shown remained genome-wide significant after adjustment for the 941 SNPs and reached P<0.05 in AoU-EUR analysis |
| Supplementary Data 19 | Proximity of single-variant genetic associations identified in AoU AFR and AoU AMR-based analysis to 12,111 previously reported common SNP associations. |
| Supplementary Data 20 | Heritability estimates for height split into 3 LD bins and the 4 MAF bins defined in Weinschtein et. al 2022, calculated using both GREML and RHEmc for comparison |
| Supplementary Data 21 | Heritability estimates for BMI, height and WHRadjBMI from RHEmc split into 3 MAF and 3 LD bins using UKBB European subset (N=343,451) |
| Supplementary Data 22 | Heritability estimates for height from RHEmc split into 17 MAF and 3 LD bins using UKBB European subset (N=343,451) with a minimum MAC=10 |
| Supplementary Data 23 | Parameters of and cumulative heritabilty estimates from exponential decay models fit with the inclusion of progressively lower MAF bins. |
| Supplementary Data 24 | Variant annotations combinations used for genomic aggregate testing |
