# Supplementary material for: Whole-genome sequencing analysis of anthropometric traits in 672,976 individuals reveals convergence between rare and common genetic associations
Source: Nat Commun. 2026 Feb 6;17:2432. doi: 10.1038/s41467-026-69208-3 (PMC12987921; doi:10.1038/s41467-026-69208-3)
Supplement: Supplementary file 4 — Reporting Summary [file 41467_2026_69208_MOESM4_ESM.pdf]

nature portfolio

Corresponding author(s): Gareth Hawkes
Andrew R Wood
Michael N Weedon

Last updated by author(s): Jul 21, 2025

Reporting Summary

Nature Portfolio wishes to improve the reproducibility of the work that we publish. This form provides structure for consistency and transparency in reporting. For further information on Nature Portfolio policies, see our Editorial Policies and the Editorial Policy Checklist.

Statistics

For all statistical analyses, confirm that the following items are present in the figure legend, table legend, main text, or Methods section.

n/a	Confirmed	
☐	☒	The exact sample size (*n*) for each experimental group/condition, given as a discrete number and unit of measurement
☒	☐	A statement on whether measurements were taken from distinct samples or whether the same sample was measured repeatedly
☐	☒	The statistical test(s) used AND whether they are one- or two-sided<br>*Only common tests should be described solely by name; describe more complex techniques in the Methods section.*
☐	☒	A description of all covariates tested
☐	☒	A description of any assumptions or corrections, such as tests of normality and adjustment for multiple comparisons
☐	☒	A full description of the statistical parameters including central tendency (e.g. means) or other basic estimates (e.g. regression coefficient) AND variation (e.g. standard deviation) or associated estimates of uncertainty (e.g. confidence intervals)
☐	☒	For null hypothesis testing, the test statistic (e.g. *F*, *t*, *r*) with confidence intervals, effect sizes, degrees of freedom and *P* value noted<br>*Give P values as exact values whenever suitable.*
☒	☐	For Bayesian analysis, information on the choice of priors and Markov chain Monte Carlo settings
☒	☐	For hierarchical and complex designs, identification of the appropriate level for tests and full reporting of outcomes
☒	☐	Estimates of effect sizes (e.g. Cohen's *d*, Pearson's *r*), indicating how they were calculated

Our web collection on statistics for biologists contains articles on many of the points above.

Software and code

Policy information about availability of computer code

Data collection	Data was not collected for this study: we used the UK Biobank and All of Us datasets.
Data analysis	Variant annotation was performed using VEP version 110. Association analysis was performed using Regenie v3.3. R-analysis was performed using v4.1.2. Plink files were generated using v1.9 and v2.0 as appropriate. The computational framework used for our WGS association analyses is available at https://doi.org/10.5281/zenodo.14204727 and plotting code is available at https://doi.org/10.5281/zenodo.18153043.

For manuscripts utilizing custom algorithms or software that are central to the research but not yet described in published literature, software must be made available to editors and reviewers. We strongly encourage code deposition in a community repository (e.g. GitHub). See the Nature Portfolio guidelines for submitting code & software for further information.

Data

Policy information about availability of data

All manuscripts must include a data availability statement. This statement should provide the following information, where applicable:

- Accession codes, unique identifiers, or web links for publicly available datasets
- A description of any restrictions on data availability
- For clinical datasets or third party data, please ensure that the statement adheres to our policy

Individual-level data cannot be shared publicly because of data availability and data return policies of the UK Biobank and All of Us. Data are available from the UK

Biobank and All of Us for researchers who meet the criteria for access (http://www.ukbiobank.ac.uk; https://allofus.nih.gov/). Genome-wide summary statistics for both the UKB-EUR and UKB-meta-analyses are available at https://zenodo.org/records/17775928.

Research involving human participants, their data, or biological material

Policy information about studies with human participants or human data. See also policy information about sex, gender (identity/presentation), and sexual orientation and race, ethnicity and racism.

Reporting on sex and gender	Findings apply to both sexes. Sex was used as a covariate in the analyses and males and females were analysed together
Reporting on race, ethnicity, or other socially relevant groupings	Principal Component Analysis of genetic data was used to define homogeneous groups based on genetic ancestry. Analysis was performed separately within each group adjusting for PCs in a mixed model to account for cryptic relatedness
Population characteristics	In UK Biobank, participants were genetically grouped into European-like, South Asian-like and African-like genetic ancestry. Participants were aged between 37 and 80 at study recruitment. In All of Us, participants were geneticlaly grouped into European-like, Admixed-American and African-American-like.
Recruitment	Participants were recruited to the UK Biobank study through self selection as described in central UK Biobank resources (http://www.ukbiobank.ac.uk), and to the All of Us study as described (https://www.researchallofus.org/)
Ethics oversight	UK Biobank protocols were approved by the National Research Ethics Service Committee. The All of Us study is overseen by the All of Us Institutional review board.

Note that full information on the approval of the study protocol must also be provided in the manuscript.

Field-specific reporting

Please select the one below that is the best fit for your research. If you are not sure, read the appropriate sections before making your selection.

☒ Life sciences ☐ Behavioural & social sciences ☐ Ecological, evolutionary & environmental sciences

For a reference copy of the document with all sections, see nature.com/documents/nr-reporting-summary-flat.pdf

Life sciences study design

All studies must disclose on these points even when the disclosure is negative.

Sample size	The study included individuals all individuals in UK Biobank who had whole genome sequences.
Data exclusions	We subset to individuals of inferred European, South-Asian and African genetic ancestry using genetic principal component analysis.
Replication	Results were replicated in the All of Us dataset.
Randomization	This study is observational, so randomisation is not applicable to this type of study
Blinding	This study is observational, so blinding is not applicable to this type of study

Reporting for specific materials, systems and methods

We require information from authors about some types of materials, experimental systems and methods used in many studies. Here, indicate whether each material, system or method listed is relevant to your study. If you are not sure if a list item applies to your research, read the appropriate section before selecting a response.

Materials & experimental systems

n/a	Involved in the study	
☒	☐	Antibodies
☒	☐	Eukaryotic cell lines
☒	☐	Palaeontology and archaeology
☒	☐	Animals and other organisms
☒	☐	Clinical data
☒	☐	Dual use research of concern
☒	☐	Plants

Methods

n/a	Involved in the study	
☒	☐	ChIP-seq
☒	☐	Flow cytometry
☒	☐	MRI-based neuroimaging

Plants

Seed stocks	NA
Novel plant genotypes	NA
Authentication	NA
